# Interactions Between BMI and Age on Fall Risk in Older Adults

**DOI:** 10.3390/epidemiologia7010018

**Published:** 2026-02-02

**Authors:** Filipe Rodrigues, Diogo Monteiro, António Miguel Monteiro, Pedro Forte

**Affiliations:** 1 ESECS—Polytechnic of Leiria, 2411-901 Leiria, Portugal; diogo.monteiro@ipleiria.pt; 2 Research Center in Sport, Health and Human Development (CIDESD), 5000-558 Vila Real, Portugal; 3 Department of Sports Sciences, Instituto Politécnico de Bragança, 5300-253 Bragança, Portugal; mmonteiro@ipb.pt (A.M.M.); pedromiguel.forte@iscedouro.pt (P.F.); 4 Research Centre for Active Living and Wellbeing (LiveWell), Instituto Politécnico de Bragança, 5300-253 Bragança, Portugal; 5 Department of Sports, Higher Institute of Educational Sciences of the Douro, 4560-708 Penafiel, Portugal

**Keywords:** functional fitness, aging, obesity paradox, sarcopenia, fall risk

## Abstract

Background/Objectives: The aging process is typically marked by a reduction in functional fitness, which heightens the likelihood of falling. Although obesity is established as a determinant of poor mobility, the interplay between excess weight and advanced age is still a topic of research interest. Therefore, this research investigated how age, sex, and Body Mass Index (BMI) interact to influence fall risk among older adults living in the community. Methods: This cross-sectional investigation involved 815 participants (M_age_ = 70.45 ± 6.10 years), stratified by age (sexagenarians, septuagenarians, octogenarians) and BMI (normal weight, overweight, obesity). Fall risk was assessed using the Timed Up and Go test. A Three-Way ANOVA examined the main and interaction effects. Results: No significant three-way interaction (*p* = 0.334) or main effect of sex (*p* = 0.079) was found. However, a significant age x BMI interaction was observed (*p* = 0.007). In sexagenarians and septuagenarians, obesity was associated with significantly slower fall risk performance compared to normal weight (*p* < 0.001). Conversely, in octogenarians, this difference was not significant (*p* = 1.000) with normal-weight individuals. Conclusions: Obesity may be a significant risk factor for falls, especially in adults aged 60 to 79 years. In octogenarians, the protective benefit of normal weight disappears, revealing a “weight paradox” likely driven by sarcopenia. Fall risk assessments and weight management strategies should be tailored to age, focusing on preserving muscle mass in octogenarians.

## 1. Introduction

The rapidly growing global population aged 65 and older presents specific challenges for public health, particularly regarding falls and loss of independence [1,2]. According to the United Nations [2], the population aged 65 and older is growing faster than any other age group due to increased life expectancy and declining fertility rates. In Portugal, this demographic shift is particularly acute. Recent census data reveal that residents aged over 65 represent approximately 23.4% of the total population, a figure expected to rise dramatically in the coming decades [3]. Although life expectancy has increased, health span has not necessarily kept pace, leading to more years being lived with disability. Aging involves a reduction in physiological reserves and functional capacity, which increases susceptibility to frailty, dependence, and non-communicable diseases [1,4]. Therefore, public health policies increasingly prioritize healthy aging, defined as the process of optimizing functional ability to support well-being in older adults [5].

A key component of this process is functional fitness, or the physical capacity to execute daily activities safely, independently, and without fatigue [6]. This construct includes muscle strength, aerobic endurance, flexibility, and agility combined with dynamic balance [7]. Agility and dynamic balance are particularly relevant given their association with falls, a primary cause of injury-related mortality and hospitalization in this demographic [8,9,10]. Although age-related declines in agility and balance are well-documented, the specific progression of these deficits and their interaction with other biological factors require further examination as described in recent research [10,11].

The decline in functional fitness is not uniform across the older adult population. It may be influenced by sociodemographic and anthropometric variables, most notably age, sex, and body composition [3,12]. Regarding sex, sexual dimorphism in physical fitness is well-documented, with males generally outperforming females in strength and speed tasks, while females often exhibit superior flexibility [13,14]. However, evidence suggests that these differences may narrow or manifest differently in advanced age groups (e.g., octogenarians), necessitating sex-stratified analyses to avoid masking specific deficit patterns [15].

Body composition, specifically Body Mass Index (BMI), also plays a critical but debated role in functional mobility. While obesity is traditionally viewed as a risk factor for functional limitation due to the increased mechanical load and metabolic dysregulation [16], it is also strongly associated with the prevalence of multimorbidity including cardiovascular diseases, diabetes, and musculoskeletal disorders (e.g., osteoarthritis), which cumulatively accelerate mobility loss [1]. However, recent studies have hinted at a “survival paradox” or complex interaction effects in the oldest-old, where low weight may also signal frailty and sarcopenia [17]. Studies conducted in Brazil and Chile [12,18] highlight how BMI and functional performance interact differently depending on socioeconomic and environmental contexts. For instance, while high BMI is generally detrimental to mobility performance [12], the protective or detrimental magnitude of weight status may shift as individuals transition from the “young-old” (60–69 years) to the “oldest-old” (80+ years).

Despite the abundance of literature on functional fitness, significant gaps remain. Previous studies conducted in Portugal have been instrumental in establishing normative standards [13] and criterion-referenced cut-offs for physical independence [19], as well as tracking temporal trends in fitness decline over the last decade [20]. However, these studies often analyze variables in isolation or focus on linear declines, potentially overlooking the interactive effects of age, sex, and BMI. For example, while extensive normative data exists for the Portuguese population [13,21], few studies have explicitly examined whether the impact of BMI on agility and fall risk is consistent across different geriatric age brackets, or if there is a critical turning point where the relationship inverts. Furthermore, recent data from specific Portuguese communities, indicates that local older adult populations may diverge from national norms [22], suggesting that regional and contemporary data are necessary to update our understanding of functional trajectories. While Rodrigues et al. [22] provided a crucial descriptive baseline for this specific community, their analysis focused primarily on main effects, potentially masking complex interdependencies. The current study seeks to overcome these limitations by explicitly modeling the Age × BMI interaction. Thereby identifying specific risk groups that main-effect analyses miss.

Moreover, international comparisons reveal discrepancies in functional fitness standards. Research involving Chinese [23], Chilean [12], and Hungarian [24] populations suggests that functional decline rates and cut-off points for independence vary significantly by culture and lifestyle, cautioning against the universal application of foreign standards without local validation. Limitations in previous study designs, such as the lack of stratification by specific BMI categories in conjunction with advanced age groups, or the reliance on cross-sectional data that may underestimate the acceleration of lower performance in the oldest-old, highlight the need for more nuanced analytical models [11,18].

The objective of this research was to examine how age, sex, and BMI mutually influence fall risk in community-dwelling older adults. By progressing beyond isolated main-effect models to investigate variable interactions, specifically the intersection of obesity and octogenarian status, this study intends to understand the epidemiological characterization of fall risk. The analysis targeted the identification of specific high-risk cohorts that broad-spectrum studies might avoid, providing data to support future research and the tailoring of public health interventions.

## 2. Materials and Methods

Sample size adequacy was assessed via a sensitivity analysis using G*Power software (v. 3.1.9.7). With a total of 815 participants distributed across 18 subgroups in a Three-Way ANOVA model (Age × BMI × Sex), the study achieved statistical power exceeding 0.80 to identify small interaction effects (f = 0.15; eta_p_^2^ = 0.02) at an alpha level of 0.05. This power level is sufficient to detect minor group differences within the proposed experimental design.

Participants were selected based on specific eligibility criteria to ensure sample homogeneity. Inclusion criteria were: (i) age between 60 and 89 years; (ii) enrollment in the community physical activity programs; and (iii) possession of medical clearance to perform physical exertion. Exclusion criteria included: (i) presence of uncontrolled chronic conditions (e.g., unstable angina, uncontrolled hypertension) that would contraindicate exercise testing; (ii) cognitive impairment (e.g., dementia) that prevented comprehension of testing instructions; (iii) dependence on assistive devices for locomotion (e.g., canes or walkers); and (iv) recent surgeries or acute musculoskeletal injuries.

The Ethics Committee of the Polytechnic of Leiria approved the research protocol (Reference No. CE/IPLEIRIA/73/2024; 24 June 2024). Employing a cross-sectional approach, data were gathered from participants enrolled in two municipal physical activity initiatives in Portugal: the “Viver Activo” program in the Municipality of Leiria [25] and the “+Idade +Saúde” [26], program in the city of Bragança. These municipalities represent distinct geographical contexts in Portugal, Leiria in the central coastal region and Bragança in the northeastern interior, encompassing a mix of urban and semi-urban community-dwelling populations. Participants were non-institutionalized older adults voluntarily seeking to maintain active lifestyles through these municipal initiatives.

The recruitment and assessment process took place during the initial registration phase at the beginning of the sports season. This timing ensured that the data reflected the participants’ baseline functional status prior to the implementation of any exercise intervention, thus eliminating potential training effects from the analysis. During registration, community-dwelling older adults were invited to participate in the study. A detailed explanation of the research objectives was provided, and it was clarified that participation was voluntary, anonymous, and entirely independent of their enrollment in the community program. Refusal to grant data usage rights for research purposes did not affect their eligibility to participate in the physical activity sessions.

Participants who agreed to collaborate provided written informed consent. Sociodemographic data and fall risk assessments were collected by trained technicians associated with the respective municipal programs, following standardized protocols. All procedures were conducted in accordance with the ethical standards of the Declaration of Helsinki for research involving human subjects.

Height assessment employed a portable stadiometer (Seca 213, Hamburg, Germany) with precision to 0.1 cm, maintaining the head in the Frankfurt plane. Weight was recorded to the nearest 0.1 kg via a digital scale (Seca 813, Hamburg, Germany), with subjects barefoot and lightly dressed. BMI was derived from the standard weight-to-height squared ratio: weight (kg)/height (m)^2^. Participants were subsequently categorized into three groups according to World Health Organization criteria: normal weight (18.5–24.9 kg/m^2^), overweight (25.0–29.9 kg/m^2^), and obesity (≥30.0 kg/m^2^). Fall risk was assessed using the TUG test, which measures agility and dynamic balance. Participants were timed as they stood up from a chair, walked 2.44 m, turned around, and returned to the chair. The best time from the two trials was recorded.

Data processing utilized IBM SPSS Statistics (v. 31.0.0.0), with significance established at *p* < 0.05. Sample characterization relied on descriptive statistics (mean and standard deviation). A Three-Way Analysis of Variance (ANOVA) assessed main and interaction effects on TUG performance, using Age, BMI, and Sex as fixed factors. The primary focus was the age × BMI interaction. Model assumptions were checked: normality via the Kolmogorov–Smirnov test and variance homogeneity via Levene’s test. Despite violations in homogeneity (*p* < 0.05), the F-statistic was deemed robust due to the large sample size [27].

Effect sizes were calculated as partial eta squared (eta_p_^2^; small = 0.01, medium = 0.06, large = 0.14) [28]. Significant interactions prompted Bonferroni-corrected pairwise comparisons to isolate group differences.

## 3. Results

The study sample comprised 815 community-dwelling older adults (Mage = 70.45 ± 6.10 years; BMI = 28.02 ± 4.16 kg/m^2^). Regarding sex distribution, the sample included 258 males (31.7%) and 557 females (68.3%). Participants were stratified into three age cohorts: 386 sexagenarians (60–69 years; 47.4%), 363 septuagenarians (70–79 years; 44.5%), and 66 octogenarians (80–89 years; 8.1%). Regarding body composition, 209 participants (25.6%) were classified as normal weight, 381 (46.7%) as overweight, and 225 (27.6%) as having obesity. The descriptive characteristics of the TUG performance, across the different age and BMI subgroups, are presented in Table 1.

A Three-Way ANOVA was conducted to examine the effects of sex, age, and BMI on fall risk. The analysis revealed no statistically significant three-way interaction between age, BMI, and sex (F(4, 797) = 1.144, *p* = 0.334, etap^2^ = 0.006). Furthermore, the main effect of sex was not significant (F(1, 797) = 3.101, *p* = 0.079), indicating that, after accounting for age and body composition, agility and dynamic balance levels were statistically comparable between men and women in this sample. Consequently, the analysis focused on the two-way interactions. A statistically significant interaction was found between age and BMI (F(4, 797) = 3.542, *p* = 0.007, etap^2^ = 0.017), suggesting that the effect of body mass index on fall risk varies across different age groups. The main effects for age (F(2, 797) = 12.94, *p* < 0.001, etap^2^ = 0.031) and BMI (F(2, 797) = 10.01, *p* < 0.001, etap^2^ = 0.025) were also significant. Detailed results are reported in Table 2.

To investigate the age × BMI interaction, Bonferroni-adjusted pairwise comparisons were performed. In the “Young-Old” groups (Sexagenarians and Septuagenarians), obesity was significantly associated with slower TUG performance compared to normal weight. Specifically, obese Sexagenarians were significantly slower than their normal-weight counterparts (*p* < 0.001), and obese Septuagenarians were significantly slower than normal-weight Septuagenarians (*p* < 0.001). However, a distinct pattern emerged in the Octogenarian group (80–89 years). In this advanced age cohort, no significant differences were found between normal weight and obese participants (*p* = 1.000). Notably, the normal-weight group exhibited a lower performance from the septuagenarian to the octogenarian stage, reaching timed up and go times (Mean = 7.69 s) comparable to, and numerically higher than, those of the obese group (Mean = 7.24 s). For details, see Table 3.

**Figure 1:**
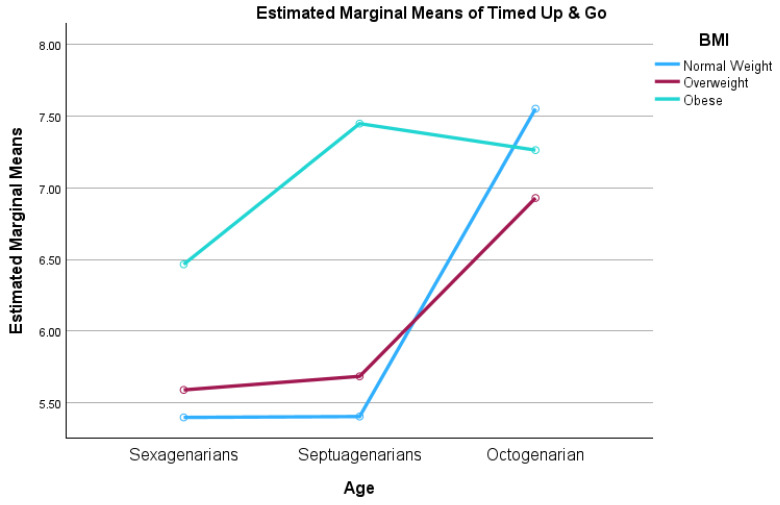
Interaction effect between age group and BMI category on fall risk. Values represent estimated marginal means.

This interaction effect is visually depicted in Figure 1, which illustrates the convergence of mobility scores between BMI groups in the oldest-old cohort. A significant crossover interaction is observed in the octogenarian group, where the performance gap between normal-weight and obese participants disappears.

## 4. Discussion

This research was designed to assess the combined influence of age, sex, and BMI on fall susceptibility in non-institutionalized older adults. The main finding of this study was the significant age × BMI interaction, which revealed distinct functional trajectories depending on the life stage. While obesity was confirmed as a significant determinant of reduced mobility in the “young-old” (sexagenarians and septuagenarians), this negative association dissipated in the “oldest-old” (octogenarians). In this advanced age group, individuals with normal weight exhibited similar functional performance compared with obese peers. These results may challenge the assumption that excess weight is the sole or primary driver of lower performance across the entire lifespan and highlight the critical, often overlooked, vulnerability of normal-weight individuals in later life.

In the cohorts of sexagenarians and septuagenarians, our data aligns with previous studies [12,18] demonstrating that obesity imposes a mechanical and metabolic constraint on mobility. The increased biomechanical load on joints and the energy cost of movement likely contribute to the significantly slower Timed Up and Go times observed in obese participants within these age brackets. This aligns with findings from diverse geographical contexts, such as the Amazonian region of Brazil [18] and Chile [12], where higher BMI consistently correlated with reduced functional fitness and agility. A key finding of this study is the ‘paradox’ observed in the octogenarian cohort. Contrary to the protective effect often attributed to normal weight in younger years, normal-weight octogenarians in our sample performed as poorly as those with obesity (*p* = 1.000). This convergence suggests a shift in the etiology of functional decline at advanced ages. While obese individuals may maintain a ‘ceiling effect’ of poor mobility due to excess mass, the decline in normal-weight individuals is likely driven by sarcopenia, the age-related loss of muscle mass and quality, and frailty [29]. Recent meta-analyses on the ‘obesity paradox’ suggest that higher weight reserves in late life may occasionally signal better muscle mass retention compared to those with lower BMI [30]. As noted in previous Portuguese studies [13,17], the preservation of muscle function is essential for autonomy. Specifically, the “normal weight” classification in octogenarians masks a pathological loss of lean muscle mass, where BMI fails to differentiate between adiposity and muscularity [31]. This finding supports the “obesity paradox” hypothesis often discussed in geriatrics, where higher weight reserves may occasionally offer a survival or functional buffer in very advanced age, or conversely, that the “healthy” normal-weight individuals are underrepresented due to survival bias [2]. BMI notoriously fails to differentiate between adiposity and muscularity in the oldest-old, meaning that our ‘normal weight’ participants may suffer from sarcopenic obesity or low lean mass, explaining their high fall risk [32]. Furthermore, it is important to acknowledge that this study applied standard WHO BMI criteria to facilitate comparison with the general population literature. However, specific geriatric recommendations (e.g., Lipschitz classification) often suggest higher normative cut-offs for older adults, viewing values up to 27 kg/m^2^ as adequate reserves. Our findings reinforce the limitation of applying general adult standards to the oldest-old, as participants classified as ‘normal weight’ by WHO standards (18.5–24.9 kg/m^2^) exhibited functional vulnerabilities that might have been flagged earlier if geriatric-specific criteria were employed.

Regarding sex differences, although females exhibited lower absolute values in raw descriptive comparisons, a pattern consistent with sexual dimorphism reported in international studies [8,14,18], the multivariate analysis revealed that Sex was not a significant main effect when adjusting for age and BMI interactions (*p* = 0.079). This suggests that the physiological mechanisms driving the decline in agility (e.g., neuromuscular deterioration and body composition changes) operate similarly in males and females, particularly regarding fall risk assessed by the TUG. This contrasts with findings from younger cohorts, where women typically outperform men in flexibility but underperform in strength [13,14], yet aligns with the notion that functional capacity tends to converge between sexes in advanced aging [8]. When placed in an international context, the functional values observed in this Portuguese sample are generally lower than the criterion-referenced standards established for the US population [16] but align more closely with recent data from Southern European [11] and South American populations [12,18]. This discrepancy reinforces the argument by Zhao et al. [11] regarding the influence of cultural, environmental, and lifestyle factors on functional norms, necessitating the use of region-specific cut-offs for accurate risk assessment.

Albeit this study benefits from a robust sample size and the use of the standardized fall risk assessment protocol, a limitation of this study is the small sample size in the octogenarian subgroups (specifically normal weight and obese males), which may reduce the statistical power for these specific pairwise comparisons. Results for these subgroups should be interpreted with caution. The cross-sectional design of this study also precludes causal inferences regarding the individual trajectory of decline. Longitudinal data would be required to confirm if normal-weight individuals indeed deteriorate at a faster rate or if this reflects a cohort effect. Additionally, body composition was assessed via BMI, which does not differentiate between fat and muscle mass, a limitation that future studies could address using bioelectrical impedance or Dual-Energy X-ray Absorptiometry (DEXA) to explicitly test the sarcopenia hypothesis in the oldest-old. Third, a potential selection bias cannot be ruled out. Although participants were assessed prior to starting the exercise programs, they were volunteers actively seeking community-based physical activities (“Viver Activo” and “+Idade +Saúde”), suggesting they may possess higher baseline motivation and functional levels than the general sedentary older adult population (healthy volunteer effect). Finally, while the sample is large, it is drawn from specific regions of Portugal and excludes nonagenarians (>90 years) to ensure statistical robustness across subgroups. Consequently, caution should be exercised when generalizing these results to the very oldest-old or to populations with different socioeconomic or cultural backgrounds. Future research should also consider controlling nutritional status and specific comorbidities that may mediate the relationship between body composition and functional decline.

## 5. Conclusions

This investigation demonstrates that falling susceptibility in non-institutionalized seniors is directed by a dynamic interaction between age and body composition. Specifically, whereas obesity significantly drives functional impairment in the ‘young-old’ cohorts (60 s and 70 s), this detrimental effect seems to be absent in the octogenarian cohort. In this advanced age group, individuals with normal weight exhibit a marked deterioration in agility and dynamic balance, performing at levels comparable to their obese peers. These results suggest that weight management guidelines and fall risk assessments should be age-adjusted rather than applied uniformly. For the “young-old,” strategies to reduce excess adiposity may remain priority interventions to preserve mobility. However, for octogenarians, the focus may shift towards preserving lean muscle mass and preventing sarcopenia, particularly among those with “normal” BMI who may be overlooked in traditional risk screenings. Future longitudinal research is needed to unravel the mechanisms underlying this “weight paradox” in advanced aging and to establish specific functional cut-offs for this growing demographic.

## Figures and Tables

**Table 1:** Sample characteristics (*n*) and descriptive statistics for the Timed Up and Go test performance (seconds), stratified by sex, age group, and BMI.

Total	Age Group	BMI Group	M	SD	*n*
	Sexagenarians	Normal weight	5.44	1.03	97
		Overweight	5.60	1.29	170
		Obese	6.64	2.04	119
		Total	5.88	1.59	386
	Septuagenarians	Normal weight	5.43	1.11	95
		Overweight	5.74	1.38	177
		Obese	7.40	3.25	91
		Total	6.07	2.12	363
	Octogenarians	Normal weight	7.69	3.12	17
		Overweight	7.01	2.82	34
		Obese	7.24	2.72	15
		Total	7.24	2.85	66
	Total	Normal weight	5.62	1.48	209
		Overweight	5.79	1.57	381
		Obese	6.99	2.66	225
		Total	6.08	1.99	815
Male	Sexagenarians	Normal weight	5.34	1.38	18
		Overweight	5.56	1.64	44
		Obese	6.17	1.21	25
		Total	5.69	1.49	87
	Septuagenarians	Normal weight	5.23	1.05	41
		Overweight	5.39	1.35	72
		Obese	7.64	4.34	34
		Total	5.87	2.53	147
	Octogenarians	Normal weight	7.22	1.08	5
		Overweight	6.71	2.07	11
		Obese	6.89	2.64	8
		Total	6.87	2.05	24
	Total	Normal weight	5.41	1.25	64
		Overweight	5.57	1.55	127
		Obese	7.00	3.34	67
		Total	5.90	2.20	258
Female	Sexagenarians	Normal weight	5.46	0.94	79
		Overweight	5.62	1.15	126
		Obese	6.76	2.20	94
		Total	5.94	1.62	299
	Septuagenarians	Normal weight	5.58	1.15	54
		Overweight	5.97	1.37	105
		Obese	7.26	2.43	57
		Total	6.21	1.78	216
	Octogenarians	Normal weight	7.89	3.69	12
		Overweight	7.15	3.15	23
		Obese	7.64	2.97	7
		Total	7.44	3.22	42
	Total	Normal weight	5.70	1.56	145
		Overweight	5.90	1.57	254
		Obese	6.98	2.32	158
		Total	6.16	1.88	557

Notes: M = Mean; SD = Standard Deviation; *n* = Number of participants. Age groups: Sexagenarians (60–69 years), Septuagenarians (70–79 years), Octogenarians (80–89 years). BMI groups: Normal weight (18.5–24.9 kg/m^2^), Overweight (25.0–29.9 kg/m^2^), and Obese (≥30.0 kg/m^2^).

**Table 2:** Summary of the Three-Way ANOVA results examining the effects of age, sex, and BMI on the Timed Up and Go performance.

Model	df	F	Sig.	eta_p_^2^
Corrected model	17	7.82	<0.001	0.14
Intercept	1	4045.92	<0.001	0.84
Sex	1	3.10	0.08	0.00
Age	2	12.94	<0.001 *	0.03
BMI	2	10.01	<0.001 *	0.02
Sex × Age	2	0.31	0.73	0.00
Sex × BMI	2	0.01	0.99	0.00
Age × BMI	4	3.54	0.01 *	0.02
Sex × age × BMI	4	1.14	0.33	0.01

Note: df = degrees of freedom; F = F-statistic; eta_p_^2^ = partial eta squared. * Significant effects (*p* < 0.05).

**Table 3:** Bonferroni-adjusted pairwise comparisons of *Timed Up and Go* (TUG) performance between BMI categories within each age group.

Age	BMI	BMI	Mean Diff.	Std. Error	*p*-Value	95% CI
Sexagenarians	Normal weight	Overweight	−0.17	0.24	1.000	[−0.74, 0.40]
	Normal weight	Obese	−1.20	0.26	<0.001 *	[−1.82, −0.59]
	Overweight	Obese	−1.04	0.22	<0.001 *	[−1.57, −0.50]
Septuagenarian	Normal weight	Overweight	−0.31	0.24	1.000	[−0.88, 0.26]
	Normal weight	Obese	−1.97	0.27	<0.001 *	[−2.63, −1.32]
	Overweight	Obese	−1.66	0.24	<0.001 *	[−2.24, −1.09]
Octogenarians	Normal weight	Overweight	0.68	0.55	1.000	[−0.65, 2.01]
	Normal weight	Obese	0.45	0.66	1.000	[−1.13, 2.04]
	Overweight	Obese	−0.23	0.58	1.000	[−1.62, 1.16]

Note: Mean Diff. = Mean Difference (seconds); CI = Confidence Interval for Difference. * Significant differences (*p* < 0.05). Comparisons are adjusted using the Bonferroni correction.

## Data Availability

The data presented in this study is available on request from the corresponding author. The data is not publicly available due to privacy and ethical restrictions regarding participant anonymity.
